# Supplementary material for: A mixed-methods systematic review of post-viral fatigue interventions: Are there lessons for long Covid?
Source: PLoS One. 2021 Nov 9;16(11):e0259533. doi: 10.1371/journal.pone.0259533 (PMC8577752; doi:10.1371/journal.pone.0259533)
Supplement: S1 File — Table 1. MEDLINE Search; Table 2. ProQuest Search. (DOCX) [file pone.0259533.s002.docx]

**S1 File**

**Search strategy for: A mixed-methods systematic review of post-viral fatigue interventions: are there lessons for long Covid?**

**Table 1. MEDLINE Search**

Interface – EBSCOhost, Research Databases, Search Screen – Advanced Search, Database - MEDLINE

Explanation of search terms used: / = MeSH Heading; asterisk (*) denotes any character; TI = title word; AB = abstract word; N3 = adjacency within three words

| S1 | (MH "Fatigue Syndrome, Chronic") |
| --- | --- |
| S2 | AB ("post viral fatigue") OR TI ("post viral fatigue") OR AB ("post viral fatigue") OR TI ("post-viral fatigue") |
| S3 | AB ("post infectious fatigue" OR "post infection fatigue" OR  "post infective fatigue") OR TI ("post infectious fatigue" OR "post infection fatigue" OR "post infective fatigue") |
| S4 | AB "chronic fatigue" OR TI "chronic fatigue" |
| S5 | AB "myalgic encephalomyelitis" OR TI "myalgic encephalomyelitis" |
| S6 | AB fibromyalgia OR TI fibromyalgia |
| S7 | AB intervention* OR TI intervention* |
| S8 | AB (trial OR trials) OR TI (trial OR trials) |
| S9 | AB "clinical trial*" OR TI "clinical trial*" |
| S10 | AB (therap* OR manage*) OR TI (therap* OR manage*) |
| S11 | AB treat* OR TI treat* |
| S12 | AB strateg* OR TI strateg* |
| S13 | AB experiment* OR TI experiment* |
| S14 | (MH "Internet-Based Intervention") |
| S15 | (MH "Early Medical Intervention") |
| S16 | (MH "Clinical Trial") |
| S17 | (MH "Early Intervention, Educational") |
| S18 | (MH "Therapeutics") |
| S19 | AB fatigue OR TI fatigue |
| S20 | AB "glandular fever" OR TI "glandular fever" |
| S21 | AB "lyme disease" OR TI "lyme disease" |
| S22 | AB (SARS OR "serious acute respiratory syndrome" OR SARSCoV) OR TI (SARS OR  "serious acute respiratory syndrome" OR SARS-CoV) |
| S23 | AB (MERS OR "middle east respiratory syndrome" OR MERSCoV) OR TI (MERS OR  "middle east respiratory syndrome" OR MERSCoV) |
| S24 | AB (covid-19 OR "covid 19") OR TI (covid-19 OR "covid 19") |
| S25 | AB H1N1 OR TI H1N1 |
| S26 | AB ebola OR TI ebola |
| S27 | AB "west nile" OR TI "west nile" |
| S28 | AB ("epstein-barr virus" OR "epstein barr" OR mononucleosis) OR TI ("epstein-barr virus" OR "epstein barr" OR mononucleosis) |
| S29 | AB (Q-fever OR "Q fever" OR "query fever") OR TI (Q-fever OR "Q fever" OR "query fever") |
| S30 | AB giardia OR TI giardia |
| S31 | AB "dengue" OR TI "dengue" |
| S32 | AB fatigue N3 infect* OR TI fatigue N3 infect* |
| S33 | (MH "Severe Acute Respiratory Syndrome") |
| S34 | (MH "Fibromyalgia") |
| S35 | (MH "Fatigue") |
| S36 | (MH "Infectious Mononucleosis") |
| S37 | (MH "Lyme Disease") |
| S38 | (MH "Middle East Respiratory Syndrome Coronavirus") |
| S39 | (MH "Ebolavirus") |
| S40 | (MH "West Nile virus") |
| S41 | (MH "West Nile Fever") |
| S42 | (MH "Q Fever") |
| S43 | (MH "Giardia") |
| S44 | (MH "Dengue") |
| S45 | S5 OR S6 OR S20 OR S21 OR S22 OR S23 OR S24 OR S25 OR S26 OR S27 OR S28 OR S29 OR S30 OR S31 OR S33 OR S34 OR S36 OR S37 OR S38 OR S39 OR S40 OR S41 OR S42 OR  S43 OR S44 |
| S46 | S19 OR S35 |
| S47 | S45 AND S46 |
| S48 | S1 OR S2 OR S3 OR S4 OR S32 |
| S49 | S7 OR S8 OR S9 OR S10 OR S11 OR S12 OR S13 OR S14 OR S15 OR S16 OR S17 OR S18 |
| S50 | S47 OR S48 |
| S51 | S49 AND S50 |

**Table 2. ProQuest Search**

Databases – APA PsycInfo

| S1 | ab("post viral fatigue") OR ti("post viral fatigue") OR ab("post-viral fatigue") OR ti("post-viral fatigue") |
| --- | --- |
| S2 | ab("post infectious fatigue") OR ab("post infection fatigue") OR ab("post infective fatigue") OR ti("post infectious fatigue") OR ti("post infection fatigue") OR ti("post infective fatigue") |
| S3 | ab("chronic fatigue") OR ti("chronic fatigue") |
| S4 | ab("myalgic encephalomyelitis") OR ti("myalgic encephalomyelitis") |
| S5 | ab(fibromyalgia) OR ti(fibromyalgia) |
| S6 | ab(intervention*) OR ti(intervention*) |
| S7 | ab(trial) OR ab(trials) OR ti(trial) OR trials |
| S8 | ab("clinical trial*") OR ti("clinical trial*") |
| S9 | ab(therap*) OR ti(therap*) OR ab(manage*) OR ti(manage*) |
| S10 | ab(treat*) OR ti(treat*) |
| S11 | ab(strateg*) OR ti(strateg*) |
| S12 | ab(experiment*) OR ti(experiment*) |
| S13 | MJMAINSUBJECT.EXACT("Chronic Fatigue Syndrome") OR MJMAINSUBJECT.EXACT("Chronic Fatigue Syndrome") |
| S14 | MJMAINSUBJECT.EXACT("Early Intervention") OR MJMAINSUBJECT.EXACT("Video-Based Interventions") OR MJMAINSUBJECT.EXACT("Mindfulness-Based Interventions") OR MJMAINSUBJECT.EXACT("Digital Interventions") OR MJMAINSUBJECT.EXACT("Workplace Intervention") |
| S15 | MJMAINSUBJECT.EXACT("Clinical Trials") OR MJMAINSUBJECT.EXACT("Randomized Clinical Trials") |
| S16 | ab(fatigue) OR ti(fatigue) |
| S17 | ab("glandular fever") OR ti("glandular fever") |
| S18 | ab("lyme disease") OR ti("lyme disease") |
| S19 | ab(SARS) OR ab("serious acute respiratory syndrome") OR ab(SARS-CoV) OR ti(SARS) OR ti("serious acute respiratory syndrome") OR ti(SARS-CoV) |
| S20 | ab(MERS) OR ab("middle east respiratory syndrome") OR ab(MERS-CoV) OR ti(MERS) OR ti("middle east respiratory syndrome") OR ti(MERS-CoV) |
| S21 | ab(covid-19) OR ab("covid 19") OR ti(covid-19) OR ti("covid 19") |
| S22 | ab(H1N1) OR ti(H1N1) |
| S23 | ab(ebola) OR ti(ebola) |
| S24 | ab("west nile") OR ti("west nile") |
| S25 | ab("epstein-barr virus") OR ab("epstein barr") OR ab(mononucleosis) OR ti("epstein-barr virus") OR ti("epstein barr") OR ti(mononucleosis) |
| S26 | ab("Q-fever") OR ab("Q fever") OR ab("query fever") OR ti("Q-fever") OR ti("Q fever") OR ti("query fever") |
| S27 | ab(giardia) OR ti(giardia) |
| S28 | ab("dengue") OR ti("dengue") |
| S29 | ab(fatigue N/3 infect*) OR ti(fatigue N/3 infect*) |
| S30 | MJMAINSUBJECT.EXACT("Severe Acute Respiratory Syndrome") |
| S31 | MJMAINSUBJECT.EXACT("Fibromyalgia") |
| S32 | MJMAINSUBJECT.EXACT("Fatigue") |
| S33 | MJMAINSUBJECT.EXACT("Lyme Disease") |
| S34 | MJMAINSUBJECT.EXACT("Middle East Respiratory Syndrome") |
| S35 | (ab("myalgic" encephalomyelitis "") OR ti("myalgic" encephalomyelitis "")) OR ab(fibromyalgia) OR ti(fibromyalgia)) OR (ab("glandular fever") OR ti("glandular fever")) OR (ab("lyme disease") OR ti("lyme disease")) OR (ab(SARS) OR ab("serious" acute respiratory syndrome "") OR ab(SARS-CoV) OR ti(SARS) OR ti("serious" acute respiratory syndrome "") OR ti(SARS-CoV)) OR (ab(MERS) OR ab("middle" east respiratory syndrome "") OR ab(MERS-CoV) OR ti(MERS) OR ti("middle" east respiratory syndrome "") OR ti(MERSCoV)) OR (ab(covid-19) OR ab("covid 19") OR ti(covid-19)  OR ti("covid 19")) OR (ab(H1N1) OR ti(H1N1)) OR (ab(ebola) OR ti(ebola)) OR (ab("west nile") OR ti("west nile")) OR (ab("epstein-barr virus") OR ab("epstein barr") OR ab(mononucleosis) OR ti("epstein-barr virus") OR ti("epstein barr") OR ti(mononucleosis)) OR (ab("Qfever") OR ab("Q" fever "") OR ab("query fever") OR ti("Qfever") OR ti("Q" fever "") OR ti("query fever")) OR (ab(giardia) OR ti(giardia)) OR  (ab("dengue") OR ti("dengue")) OR (ab(fatigue NEAR/3 infect*) OR ti(fatigue NEAR/3  infect*)) OR MJMAINSUBJECT.EXACT("Severe Acute Respiratory Syndrome") OR  MJMAINSUBJECT.EXACT("Fibromyalgia") OR MJMAINSUBJECT.EXACT("Fatigue") OR  MJMAINSUBJECT.EXACT("Lyme Disease") OR MJMAINSUBJECT.EXACT("Middle East Respiratory Syndrome") |
| S36 | (ab(fatigue) OR ti(fatigue)) OR MJMAINSUBJECT.EXACT("Fatigue") |
| S37 | ((ab("myalgic" encephalomyelitis "") OR ti("myalgic" encephalomyelitis "")) OR ab(fibromyalgia) OR ti(fibromyalgia)) OR (ab("glandular fever") OR ti("glandular fever")) OR (ab("lyme disease") OR ti("lyme disease")) OR (ab(SARS) OR ab("serious" acute respiratory syndrome "") OR ab(SARS-CoV) OR ti(SARS) OR ti("serious" acute respiratory syndrome "") OR ti(SARS-CoV)) OR (ab(MERS) OR ab("middle" east respiratory syndrome "") OR ab(MERS-CoV) OR ti(MERS) OR ti("middle" east respiratory syndrome "") OR ti(MERSCoV)) OR (ab(covid-19) OR ab("covid 19") OR ti(covid-19) OR ti("covid 19")) OR (ab(H1N1) OR ti(H1N1)) OR (ab(ebola) OR ti(ebola)) OR (ab("west nile") OR ti("west nile")) OR (ab("epstein-barr virus") OR ab("epstein barr") OR ab(mononucleosis) OR ti("epstein-barr virus") OR ti("epstein barr") OR ti(mononucleosis)) OR (ab("Qfever") OR ab("Q" fever "") OR ab("query fever") OR ti("Qfever") OR ti("Q" fever "") OR ti("query fever")) OR (ab(giardia) OR ti(giardia)) OR  (ab("dengue") OR ti("dengue")) OR (ab(fatigue NEAR/3 infect*) OR ti(fatigue NEAR/3  infect*)) OR MJMAINSUBJECT.EXACT("Severe Acute Respiratory Syndrome") OR  MJMAINSUBJECT.EXACT("Fibromyalgia") OR MJMAINSUBJECT.EXACT("Fatigue") OR  MJMAINSUBJECT.EXACT("Lyme Disease") OR MJMAINSUBJECT.EXACT("Middle East Respiratory Syndrome")) AND ((ab(fatigue) OR ti(fatigue)) OR MJMAINSUBJECT.EXACT("Fatigue")) |
| S38 | (ab("post viral fatigue") OR ti("post viral fatigue") OR ab("post-viral fatigue") OR  ti("post-viral fatigue")) OR (ab("post infectious fatigue") OR ab("post infection fatigue")  OR ab("post infective fatigue") OR ti("post infectious fatigue") OR ti("post infection fatigue") OR ti("post infective fatigue")) OR (ab("chronic fatigue") OR ti("chronic fatigue")) OR (ab(fatigue NEAR/3 infect*) OR ti(fatigue NEAR/3 infect*)) |
| S39 | (ab(intervention*) OR ti(intervention*)) OR (ab(trial) OR ab(trials) OR ti(trial) OR trials) OR (ab("clinical trial*") OR ti("clinical trial*")) OR (ab(therap*) OR ti(therap*) OR ab(manage*) OR ti(manage*)) OR (ab(treat*) OR ti(treat*)) OR (ab(strateg*) OR ti(strateg*)) OR (ab(experiment*) OR ti(experiment*)) |
| S40 | (((ab("myalgic" encephalomyelitis "") OR ti("myalgic" encephalomyelitis "")) OR (ab(fibromyalgia) OR ti(fibromyalgia)) OR (ab("glandular fever") OR ti("glandular fever")) OR (ab("lyme disease") OR ti("lyme disease")) OR (ab(SARS) OR ab("serious"  acute respiratory syndrome "") OR ab(SARS-CoV) OR ti(SARS) OR ti("serious" acute  respiratory syndrome "") OR ti(SARS-CoV)) OR (ab(MERS) OR ab("middle" east  respiratory syndrome "") OR ab(MERS-CoV) OR ti(MERS) OR ti("middle" east respiratory  syndrome "") OR ti(MERSCoV)) OR (ab(covid-19) OR ab("covid 19") OR ti(covid-19)  OR ti("covid 19")) OR (ab(H1N1) OR ti(H1N1)) OR (ab(ebola) OR ti(ebola)) OR (ab("west nile") OR ti("west nile")) OR (ab("epstein-barr virus") OR ab("epstein barr") OR ab(mononucleosis) OR ti("epstein-barr virus") OR ti("epstein barr") OR ti(mononucleosis)) OR (ab("Qfever") OR ab("Q" fever "") OR ab("query fever") OR ti("Qfever") OR ti("Q" fever "") OR ti("query fever")) OR (ab(giardia) OR ti(giardia)) OR  (ab("dengue") OR ti("dengue")) OR (ab(fatigue NEAR/3 infect*) OR ti(fatigue NEAR/3  infect*)) OR MJMAINSUBJECT.EXACT("Severe Acute Respiratory Syndrome") OR  MJMAINSUBJECT.EXACT("Fibromyalgia") OR MJMAINSUBJECT.EXACT("Fatigue") OR  MJMAINSUBJECT.EXACT("Lyme Disease") OR MJMAINSUBJECT.EXACT("Middle East Respiratory Syndrome")) AND ((ab(fatigue) OR ti(fatigue)) OR MJMAINSUBJECT.EXACT("Fatigue"))) OR ((ab("post viral fatigue") OR ti("post viral  fatigue") OR ab("post-viral fatigue") OR ti("post-viral fatigue")) OR (ab("post infectious fatigue") OR ab("post infection fatigue") OR ab("post infective fatigue") OR ti("post infectious fatigue") OR ti("post infection fatigue") OR ti("post infective fatigue")) OR  (ab("chronic fatigue") OR ti("chronic fatigue")) OR (ab(fatigue NEAR/3 infect*) OR ti(fatigue NEAR/3 infect*))) |
| S41 | ((ab(intervention*) OR ti(intervention*)) OR (ab(trial) OR ab(trials) OR ti(trial) OR trials) OR (ab("clinical trial*") OR ti("clinical trial*")) OR (ab(therap*) OR ti(therap*) OR ab(manage*) OR ti(manage*)) OR (ab(treat*) OR ti(treat*)) OR (ab(strateg*) OR  ti(strateg*)) OR (ab(experiment*) OR ti(experiment*))) AND ((((ab("myalgic" encephalomyelitis "") OR ti("myalgic" encephalomyelitis "")) OR (ab(fibromyalgia) OR  ti(fibromyalgia)) OR (ab("glandular fever") OR ti("glandular fever")) OR (ab("lyme disease") OR ti("lyme disease")) OR (ab(SARS) OR ab("serious" acute respiratory syndrome "") OR ab(SARS-CoV) OR ti(SARS) OR ti("serious" acute respiratory syndrome "") OR ti(SARS-CoV)) OR (ab(MERS) OR ab("middle" east respiratory syndrome "") OR  ab(MERS-CoV) OR ti(MERS) OR ti("middle" east respiratory syndrome "") OR ti(MERSCoV)) OR (ab(covid-19) OR ab("covid 19") OR ti(covid-19) OR ti("covid 19")) OR  (ab(H1N1) OR ti(H1N1)) OR (ab(ebola) OR ti(ebola)) OR (ab("west nile") OR ti("west  nile")) OR (ab("epstein-barr virus") OR ab("epstein barr") OR ab(mononucleosis) OR  ti("epstein-barr virus") OR ti("epstein barr") OR ti(mononucleosis)) OR (ab("Qfever")  OR ab("Q" fever "") OR ab("query fever") OR ti("Qfever") OR ti("Q" fever "") OR  ti("query fever")) OR (ab(giardia) OR ti(giardia)) OR (ab("dengue") OR ti("dengue"))  OR (ab(fatigue NEAR/3 infect*) OR ti(fatigue NEAR/3 infect*)) OR MJMAINSUBJECT.EXACT("Severe Acute Respiratory Syndrome") OR MJMAINSUBJECT.EXACT("Fibromyalgia") OR MJMAINSUBJECT.EXACT("Fatigue") OR  MJMAINSUBJECT.EXACT("Lyme Disease") OR MJMAINSUBJECT.EXACT("Middle East Respiratory Syndrome")) AND ((ab(fatigue) OR ti(fatigue)) OR MJMAINSUBJECT.EXACT("Fatigue"))) OR ((ab("post viral fatigue") OR ti("post viral  fatigue") OR ab("post-viral fatigue") OR ti("post-viral fatigue")) OR (ab("post infectious fatigue") OR ab("post infection fatigue") OR ab("post infective fatigue") OR ti("post infectious fatigue") OR ti("post infection fatigue") OR ti("post infective fatigue")) OR  (ab("chronic fatigue") OR ti("chronic fatigue")) OR (ab(fatigue NEAR/3 infect*)  OR ti(fatigue NEAR/3 infect*)))) |
