# Supplementary material for: A mixed-methods systematic review of post-viral fatigue interventions: Are there lessons for long Covid?
Source: PLoS One. 2021 Nov 9;16(11):e0259533. doi: 10.1371/journal.pone.0259533 (PMC8577752; doi:10.1371/journal.pone.0259533)
Supplement: S2 File — (DOCX) [file pone.0259533.s003.docx]

**S2 File**

11 different self-report scales were used to measure fatigue in the studies assessed in the statistical analysis. All instruments reported in these papers were subjective/self-report scales - there were no objective or physiological measures reported - raising questions around accuracy and consistency as people with fatigue may not self-assess well. Table x compares the characteristics of the self-report fatigue scales used by studies in the statistical analysis.

**Table 1. Comparison of fatigue measurement scales**

| **Fatigue measure** | **Purpose** | **Content/Constructs** | **Scoring** | **Functional disability** |
| --- | --- | --- | --- | --- |
| Chalder fatigue scale (11-item). | To assess the severity of fatigue in general practice settings. | Seven items related to physical symptoms of fatigue (e.g., Do you feel weak? Do you need to rest more?). Four items related to mental symptoms of fatigue (e.g., Do you have difficulty concentrating? How is your memory?). | Scoring using a bimodal response system or a Likert score with weights assigned to each response choice. This measure uses either Likert rating scales with 4 response options, or bimodal responses: ‘Yes’ meaning positive for this symptom and scoring 1 point, and ‘No’ meaning negative for this symptom and scoring 0 point. Score range is 0–11 for bimodal response format; 0–33 for Likert Scale. | The items related to physical fatigue would appear to describe functional disability. |
| Fatigue questionnaire scale (FS-14) | The fatigue questionnaire scale (FS-14) is used to  evaluate the fatigue level of patients. | FS-14 consists of 14 items, including 6 items reflecting mental fatigue and 8 items reflecting physical fatigue. | Scoring using a bimodal response system or a Likert score with weights assigned to each response choice. This measure uses either Likert rating scales with 4 response options, or bimodal responses: ‘Yes’ meaning positive for this symptom and scoring 1 point, and ‘No’ meaning negative for this symptom and scoring 0 point. Score range is 0–14 for bimodal response format; 0–33 for Likert Scale. | The items related to physical fatigue would appear to describe functional disability. |
| Checklist Individual Strength (CIS) – fatigue severity subscale | CIS was intended to be a questionnaire to test the “behavioural, emotional, social, and cognitive aspects” of ME/CFS and to identify the multiple dimensions of ME/CFS patients’ disability. | 20 items relating to subjective fatigue, concentration, motivation, physical activity – for studies of fatigue, often only the subjective experience of fatigue dimension is used. | Items are rated on a seven-point scale. For the subjective experience of fatigue dimension, the score range is 8–56. Higher scores indicate more fatigue. A cut-off point of 35 on the subjective experience of fatigue dimension is usually used to define a clinical level of fatigue. | The subscale fatigue severity has been shown to be correlated with activity levels measured by an Actometer and so could be thought to assess functional disability. |
| Fatigue severity scale (FSS) | To measure fatigue severity in a manner that facilitates research in the experience of fatigue in a variety of medical and neurologic disorders | Nine statements concerning  respondent’s fatigue, e.g., how fatigue affects motivation, exercise, physical functioning, carrying out duties, interfering with work, family, or social life. | Scale is a 7-point Likert scale where 1 = Strongly Disagree and 7 = Strongly  Agree. Sum responses and divide by number of items for scale score.  Score range is 1–7, though sometimes raw scores are reported. | Items are related to the consequences of fatigue. |
| Global fatigue index (GFI) - Measured within the Multidimensional Assessment of Fatigue (MAF) questionnaire | The MAF was originally developed to measure self-reported fatigue in adults with rheumatoid arthritis (RA) but subsequently has  been used to measure fatigue in adults with other  chronic conditions. | The MAF is a self-administered  questionnaire to measure 4 dimensions of self-reported  fatigue: degree and severity, amount of distress it causes, its timing (how often it occurs and if it changed over the past week), and the degree to which fatigue interferes with activities of daily living. | Numerical rating scale (1–10) for items 1, and 4–14 (1 = not at all, 10 = a great deal), item 2 (1 = mild to 10 = severe), item 3 (1 = no distress, 10 = a great deal of distress), Categorical response (1–4) for Timing items 15 and 16. For GFI, score range is 1–50 (1 = no fatigue, 50 = severe fatigue). | Higher scores indicate more severe fatigue, fatigue distress, or interference with activities of daily living. |
| Fatigue Assessment Instrument (FAI) | The FAI was developed in order to assess fatigue across a range of medical conditions and has been validated in different diagnoses. It may be valuable for screening individuals in clinical practice and may also be useful for research endeavours. | It has four sub-scores: severity, specificity, consequences of fatigue and responsiveness to rest/sleep. | The fatigue severity score is the mean of 11 items among the 29 constituting the FAI, quoted between 1 and 7 by the patient, 1 representing a total disagreement and 7 representing a total agreement with the written statements. Higher scores are indicative of greater problems with fatigue. | Some of the questions in the FAI relate to physical functioning and so will give some indication of an individual’s physical disability |
| FIQ VAS Fatigue scale (0-100) | To quantify global fibromyalgia severity and identify patients with significant symptoms of fatigue, poor sleep, depression or anxiety with brevity, enabling rapid patient assessment and informing treatment decisions in busy clinics. | FIQ VASs performed as well as the full FIQ in assessing global disease severity, and cut-off scores on individual FIQ VASs could be established to identify patients with significant symptoms of fatigue, poor sleep and depression | The VAS for fatigue included in the Fibromyalgia Impact  Questionnaire (FIQ) is a one-dimensional measure of fatigue, ranging from 0 mm (no tiredness) to 100 mm (very tired). | The fatigue scale on the FIQ VAS is one-dimensional focusing on how tired an individual has felt and therefore may not capture physical disability. |
| Multidimensional fatigue inventory (MFI-20) | The Multidimensional Fatigue Inventory (MFI) is a 20-item self-report instrument designed to measure fatigue. | The MFI is a 20-item scale designed to evaluate five dimensions of fatigue: general  fatigue, physical fatigue, reduced motivation, reduced activity, and mental fatigue. | Respondents use a scale ranging from 1 to 7 to indicate how aptly certain statements regarding fatigue represent their experiences. Several positively phrased items are reverse-scored. Higher total scores correspond with more acute levels of fatigue. | Some of the questions in the MFI-20 relate to physical functioning and so will give some indication of an individual’s physical disability |
| Profile of fatigue related states (PFRS) | To measure symptomology specifically related to ME/CFS. | Each item lists a symptom typical of ME/CFS and respondents are asked to indicate how intensely they have experienced that symptom over the past week. | Responses are given in a seven-point Likert-scale format ranging from 0 (not at all) through 3 (moderately) to 6 (extremely). Average item scores are then computed for four separate factors: Emotional Distress, Fatigue, Cognitive Difficulty, and Somatic symptoms – though in F021 scores are given as total. | Unclear how sensitive this scale is to functional disability. |
| Brief fatigue inventory's (BFI) | To assess the severity of fatigue and the impact of fatigue on daily functioning | Severity of fatigue and the impact of fatigue on daily functioning in the past 24 hours. The six interference items (general activity, mood, walking ability, normal work, relations with other people, enjoyment of life) correlate with standard quality-of-life measures. | This symptom assessment tool measures nine items on 10-point numeric scales for fatigue level and interference with daily life. A global fatigue score can be obtained by averaging all the items on the BFI. Cut points for fatigue level suggested are 1–3 (mild), 4–7 (moderate), and 8–10 (severe) (Chang, 2007). Levels of 4 or greater suggest a need for intervention beyond activities for prevention. | Some of the questions in the BFI relate to physical functioning and so will give some indication of an individual’s physical disability |
| Functional assessment of chronic fatigue (FACIT-F) | To assess specific quality of life concerns related to fatigue in cancer patients. The FACIT-F was originally developed to assess the fatigue associated with anemia. | Designed to assess fatigue/ tiredness and its impact on daily activities and functioning in a number of chronic diseases. The instrument includes items such as tiredness, weakness, listlessness, lack of energy, and the impact of these feelings on daily functioning (e.g., sleeping, and social activities). | The level of fatigue is measured on a five-point Likert-type scale (0 = not at all; 1 = a little bit; 2 = somewhat; 3 = quite a bit; and 4 = very much). All items contribute to the sum score with equal weight. The scale range is 0 to 52, with 0 being the worst possible score and 52 being the best possible score indicating no fatigue - *however, F035 scored this the opposite way, i.e. total score ranges from 0 (no fatigue) to 52 (maximum degree of fatigue). | Some of the questions in the FACIT-F relate to physical functioning and so will give some indication of an individual’s physical disability |
