# Supplementary material for: A mixed-methods systematic review of post-viral fatigue interventions: Are there lessons for long Covid?
Source: PLoS One. 2021 Nov 9;16(11):e0259533. doi: 10.1371/journal.pone.0259533 (PMC8577752; doi:10.1371/journal.pone.0259533)
Supplement: S3 File — (DOCX) [file pone.0259533.s004.docx]

**S3 File**

**Table 1. Overview of intervention effectiveness using standardised measures, including follow-up assessments.**

|  |  |  | **Post-intervention** | | | | **Follow-up** | | | |
| --- | --- | --- | --- | --- | --- | --- | --- | --- | --- | --- |
| **Author/year** | **Control/Intervention Groups** | **Baseline fatigue severity (%)** | **Change in fatigue from baseline (%)** | **Mean difference (95% CI)** | **Sig.** | **SMD** | **Change in fatigue from baseline (%)** | **Mean difference (95% CI)** | **Sig.** | **SMD** |
| O'Dowd (2006) | SMC | 72.4 | -6.4 |  |  |  |  |  |  |  |
|  | CBT | 75.8 | -21.5 | -11.8 [-20.8, -2.8] | 0.027 | 0.503 |  |  |  |  |
|  | GET | 75.5 | -10.6 | -1.2 [-9.8, 7.3] | 0.957 | 0.055 |  |  |  |  |
| Wiborg (2015) | Waiting list | 87.3 | -6.9 |  |  |  |  |  |  |  |
|  | CBT (large group) | 90.4 | -36.9 | -26.9 [-33.1, -20.6] | <0.001 | 1.126 |  |  |  |  |
|  | CBT (small group) | 88.5 | -35.6 | -27.5 [-33.7, -21.3] | <0.001 | 1.170 |  |  |  |  |
| Hansen (2013) | Healthy controls | 34.8 | 0.0 |  |  |  |  |  |  |  |
|  | CBT/GET/Group therapy | 76.8 | -47.4 | -5.4 [-17.7, 6.9]^¥^ | 0.398^¥^ | 0.278^¥^ |  |  |  |  |
| Heald (2019) | Waiting list | 78.9 | -1.6 |  |  |  |  |  |  |  |
|  | Group CBT | 77.3 | -23.7 | -23.7 [-29.2, -18.2] | <0.001 | 2.266 |  |  |  |  |
| Kim (2015) | SMC | 58.0 | -0.2 |  |  |  | -3.3 |  |  |  |
|  | Acupuncture + SMC | 61.2 | -21.5 | -18.2 [-23.5, -12.8] | <0.001 | 0.984 | -22.2 | -15.7 [-21.5, -9.8] | <0.001 | 0.773 |
|  | Sa-am Acupuncture + SMC | 62.5 | -19.5 | -14.8 [-20.4, -9.2] | <0.001 | 0.768 | -20.7 | -12.8 [-18.7, -7.0] | <0.001 | 0.629 |
| Mist (2018) | Group Education | 70.6 | -1.2 |  |  |  | 4.9 |  |  |  |
|  | Group Acupuncture | 72.9 | -23.9 | -20.4 |  |  | -28.0 | -30.6 |  |  |
| Ng (2013) | Sham Acupuncture | 85.2 | -16.4 |  |  |  |  |  |  |  |
|  | Acupuncture | 91.6 | -27.5 | -4.6 [-8.3, -0.9] | 0.016 | 0.204 |  |  |  |  |
| Shu (2016) | Acupuncture (Healthy Controls) | 45.5 | -15.0 |  |  |  |  |  |  |  |
|  | Acupuncture | 68.7 | -23.9 | 14.4 |  |  |  |  |  |  |
|  | Moxibustion | 66.2 | -30.0 | 5.7 |  |  |  |  |  |  |
| UÄŸurlu (2017) | Sham Acupuncture | 89.4 | -11.9 |  |  |  | -24.4 |  |  |  |
|  | Acupuncture | 85.7 | -15.1 | -7.0 [-13.1, -0.8] | 0.077 | 0.512 | -33.4 | -9.0 [-17.0, -1.1] | 0.088 | 0.493 |
| Kim (2013) | Sham Moxibustion | 60.0 | -4.3 |  |  |  |  |  |  |  |
|  | Moxibustion | 59.8 | -14.4 | -10.3 |  |  |  |  |  |  |
| Clark (2017) | SMC | 78.8 | -9.4 |  |  |  |  |  |  |  |
|  | GET + SMC | 79.7 | -21.8 | -11.5 [-17.6, -5.4] | <0.001 | 0.524 |  |  |  |  |
| Ericsson (2016) | Relaxation | 81.8 | -5.5 |  |  |  |  |  |  |  |
|  | Group-based GET | 81.4 | -8.6 | -3.5 |  |  |  |  |  |  |
| Oka (2014) | Pharma | 79.1 | -0.9 |  |  |  |  |  |  |  |
|  | Yoga + Pharma | 78.5 | -20.3 | -20.0 [-34.6, -5.4] | 0.012 | 0.978 |  |  |  |  |
| Windhorst (2017) | GET | 61.0 | -15.3 |  |  |  |  |  |  |  |
|  | Biofeedback | 51.9 | -16.6 | -10.5 [-38.3, 17.3] | 0.077 | 0.486 |  |  |  |  |
| Weatherley-Jones (2004) | Placebo | 77.1 | -10.2 |  |  |  |  |  |  |  |
|  | Homeopathic Medication | 77.8 | -14.5 | -3.7 [-4.9, -2.5] | <0.001 | 0.855 |  |  |  |  |
| Dailey (2013) | No TENS | 52.0 | -2.0 |  |  |  |  |  |  |  |
|  | Active TENS | 50.0 | -6.0 | -6.0 [-16.9, 4.9] | 0.535 | 0.239 |  |  |  |  |
|  | Placebo TENS | 50.0 | 5.0 | 5.0 [-6.5, 16.5] | 0.647 | -0.189 |  |  |  |  |
| Fitzgibbon (2018) | Sham rTMS | 68.3 | -8.0 |  |  |  | -7.3 |  |  |  |
|  | rTMS | 73.3 | -13.6 | -0.6 |  |  | -28.3 | -16.0 |  |  |
| Van Hoof (2003) | No infection (with HBOT) | 76.0 | -4.1 |  |  |  | -3.3 |  |  |  |
|  | Mycoplasma hominis infection (with HBOT) | 74.9 | -4.4 | -1.4 [-3.5, 0.8] | 0.215 | 0.367 | -2.1 | 0.0 [-2.9, 2.9] | 1 | 0 |
| Perrin (2011) | Healthy controls | 4.3 | -0.9 |  |  |  |  |  |  |  |
|  | CFS patients (osteopathic treatment) | 50.0 | -18.2 | 28.4 [-4.6, 61.4]^¥^ | 0.0013^¥^ | -1.827^¥^ |  |  |  |  |
|  | CFS patients (any treatment) | 39.8 | 5.9 | 42.3 [33.2, 51.3]^¥^ | <0.001^¥^ | -4.313^¥^ |  |  |  |  |
| Keijmel (2017) | Placebo | 87.9 | -25.8 |  |  |  |  |  |  |  |
|  | Medication | 90.4 | -22.1 | 6.3 [3.7, 8.8] | <0.001 | -0.752 |  |  |  |  |
|  | CBT | 86.9 | -37.7 | -12.9 [-15.1, -10.8] | <0.001 | 1.784 |  |  |  |  |
| Racine (2019) | Control | 67.4 | 1.4 |  |  |  |  |  |  |  |
|  | Operant Learning | 64.1 | -5.9 | -10.6 [-22.5, 1.3] | 0.155 | 0.520 |  |  |  |  |
|  | Energy Conservation | 68.3 | 0.5 | 0.0 [-9.6, 9.6] | - | - |  |  |  |  |
| Ridsdale (2012) | SMC + CBT Booklet | 70.9 | -24.5 |  |  |  | -29.1 |  |  |  |
|  | GET | 75.2 | -30.9 | -2.1 [-10.2, 6.0] | 0.855 | 0.084 | -31.2 | 2.1 [-5.5, 9.7] | 0.8583 | -0.09 |
|  | Counselling | 75.2 | -26.1 | 2.7 [-5.1, 10.6] | 0.784 | -0.111 | -29.1 | 4.2 [-3.5, 12.0] | 0.5167 | -0.173 |
| White (2011) | SMC | 85.8 | -13.0 |  |  |  | -13.6 |  |  |  |
|  | SMC + APT | 86.4 | -14.5 | -0.9 [-5.5, 3.7] | 0.983 | 0.043 | -16.4 | -2.1 [-6.8, 2.5] | 0.835 | 0.1 |
|  | SMC + CBT | 83.9 | -18.8 | -7.6 [-12.5, -2.7] | 0.011 | 0.339 | -22.4 | -10.6 [-15.5, -5.7] | <0.001 | 0.476 |
|  | SMC + GET | 85.5 | -19.7 | -7.0 [-11.6, -2.3] | 0.024 | 0.328 | -23.0 | -9.7 [-14.4, -5.0] | <0.001 | 0.453 |
| Sharpe (2015) | SMC | 85.8 | -24.5 |  |  |  |  |  |  |  |
|  | SMC + APT | 86.4 | -24.2 | 0.9 [-5.7, 7.5] | 0.993 | -0.035 |  |  |  |  |
|  | SMC + CBT | 83.9 | -28.2 | -5.5 [-12.1, 1.2] | 0.362 | 0.210 |  |  |  |  |
|  | SMC + GET | 85.5 | -27.6 | -3.3 [-9.7, 3.0] | 0.742 | 0.133 |  |  |  |  |
| Vos-Vromans (2016) | CBT | 89.7 | -31.0 |  |  |  | -22.9 |  |  |  |
|  | Multidisciplinary rehabilitation | 90.6 | -37.6 | -5.7 [-13.6, 2.2] | 0.158 | 0.197 | -36.7 | -12.9 [-20.8, -5.1] | 0.001 | 0.454 |
| Raijmakers (2019) | Placebo | 87.9 | -27.3 |  |  |  |  |  |  |  |
|  | Medication | 90.4 | -21.0 | 8.7 [6.4, 11.1] | <0.001 | -1.120 |  |  |  |  |
|  | CBT | 86.9 | -21.3 | 5.0 [2.7, 7.3] | <0.001 | -0.670 |  |  |  |  |
| Fernie (2016) | CBT | 69.6 | -21.5 |  |  |  | -25.0 |  |  |  |
|  | GET | 68.2 | -14.5 | 5.6 [-2.9, 14.1] | 0.199 | -0.224 | -21.3 | 2.3 [-5.3, 9.8] | 0.568 | -0.096 |
| Stubhaug (2008) | Placebo | 77.4 | -5.6 |  |  |  |  |  |  |  |
|  | Medication | 75.0 | -6.2 | -3.0 [-6.8, 0.7] | 0.349 | 0.447 |  |  |  |  |
|  | CBT | 74.6 | -10.9 | -8.2 [-13.2, -3.1] | 0.001 | 0.916 |  |  |  |  |
| Lee (2015) | Waiting List | 65.0 | -6.7 |  |  |  |  |  |  |  |
|  | Oriental medicine music therapy | 60.0 | -23.3 | -21.7 [-30.4, -12.9] | <0.001 | 1.237 |  |  |  |  |
| El Mokadem (2020) | Waiting List | 82.3 | -7.7 |  |  |  |  |  |  |  |
|  | Three Principles | 81.0 | -45.4 | -39.0 [-51.8, -26.2] | <0.001 | 1.670 |  |  |  |  |
|  |  |  |  |  |  |  |  |  |  |  |
| Maddali (2016) | Educational course | 32.9 | -0.9 |  |  |  |  |  |  |  |
|  | Tai Ji Quan | 39.5 | -7.9 | -0.4 [-9.6, 8.9] | 0.933 | 0.023 |  |  |  |  |
| Jason (2010) | Waiting list | 90.7 | 2.6 |  |  |  |  |  |  |  |
|  | Social support | 93.9 | -12.6 | -12.0 [-20.7, -3.4] | 0.014 | 0.826 |  |  |  |  |
| Wearden (2010) | SMC | 31.3 | -3.1 |  |  |  | -2.6 |  |  |  |
|  | Pragmatic rehab | 31.8 | -6.4 | -2.8 [-5.7, 0.1] | 0.112 | 0.270 | -5.4 | -2.3 [-5.0, 0.4] | 0.2254 | 0.236 |
|  | Supportive listening | 31.9 | -2.6 | 1.1 [-1.4, 3.6] | 0.703 | -0.117 | -3.4 | -0.3 [-2.8, 2.2] | 0.9883 | 0.03 |
| Marques (2014) | SMC | 81.3 | 0.7 |  |  |  |  |  |  |  |
|  | Self-regulation based physical activity | 79.2 | -7.0 | -9.9 [-16.3, -3.4] | <0.001 | 0.513 |  |  |  |  |
| Friedburg (2016) | SMC | 93.7 | -3.3 |  |  |  | -3.3 |  |  |  |
|  | Self-management & web diaries | 92.0 | -6.7 | -5.0 [-9.1, -0.9] | 0.048 | 0.434 | -8.7 | -7.0 [-11.8, -2.2] | 0.014 | 0.514 |
|  | Self-management & paper diaries | 90.8 | -8.8 | -8.3 [-12.4, -4.3] | 0.000 | 0.724 | -5.8 | -5.3 [-10.2, -0.5] | 0.0824 | 0.391 |
| Marques (2017) | SMC | 81.3 | 3.2 |  |  |  |  |  |  |  |
|  | Self-regulation based physical activity | 79.2 | -8.4 | -13.7 [-20.1, -7.3] | <0.001 | 0.720 |  |  |  |  |
| Tummers (2010) | SMC | 87.3 | -31.3 |  |  |  |  |  |  |  |
|  | Self-instruction & CBT | 85.6 | -29.2 | 0.4 [-6.2, 7.1]^ǂ^ | 0.883^ǂ^ | -0.015^ǂ^ |  |  |  |  |

P < 0.05, SMD = Standardised mean difference (Cohen’s d and Hedges’ g), ǂ = non-inferiority trial to see if one treatment is no less effective than the other, ¥ = fatigued individuals vs healthy controls, SMC = Standard Medical Care, Cognitive behavioural therapy = CBT, Graded exercise therapy = GET, Adaptive pacing therapy = APT, 95% CI = 95% Confidence intervals.
