# Supplementary material for: A mixed-methods systematic review of post-viral fatigue interventions: Are there lessons for long Covid?
Source: PLoS One. 2021 Nov 9;16(11):e0259533. doi: 10.1371/journal.pone.0259533 (PMC8577752; doi:10.1371/journal.pone.0259533)
Supplement: Attachment — Submitted filename: Response to Reviewer 2 12.10.21.docx [file pone.0259533.s006.docx]

Response to Reviewer #2 Stage 2 4/10/21

Thanks for the final clarification you requested;

1. You have included quality appraisal using the MMAT. The scores and interpretation should be mentioned. And since it is score, I don’t think that * is needed in the table 1.

All asterisks have been removed

2. Please revise the title of Table 2.

Thanks, we have amended the title of the table

3	You report fatigue severity scores in a common metric scale. I still think that it would be to	address the fact that the fatigue was measured using different scores – how many different scales were there, which were the most commonly used?

We have included a supplementary file 2 that addresses the range of measures and this is referred to on line 219 of page 9 but we have added a small amount of detail to identify the most commonly used in the following section

Finally

We noted that the abstract and the conclusion were not quite the same and so we have slightly amended the abstract to ensure the same message
