# Supplementary material for: A mixed-methods systematic review of post-viral fatigue interventions: Are there lessons for long Covid?
Source: PLoS One. 2021 Nov 9;16(11):e0259533. doi: 10.1371/journal.pone.0259533 (PMC8577752; doi:10.1371/journal.pone.0259533)
Supplement: Attachment — Submitted filename: Response to Reviewer Comments.docx [file pone.0259533.s005.docx]

**Response to Reviewers**

Additional journal requirements.

1. Please ensure that your manuscript meets PLOS ONE's style requirements, including those for file naming. The PLOS ONE style templates can be found at

<https://journals.plos.org/plosone/s/file?id=wjVg/PLOSOne_formatting_sample_main_body.pdf>

and

<https://journals.plos.org/plosone/s/file?id=ba62/PLOSOne_formatting_sample_title_authors_affiliations.pdf>

We have corrected the formatting according to the journal requirements

2. We note that you have stated that you will provide repository information for your data at acceptance. Should your manuscript be accepted for publication, we will hold it until you provide the relevant accession numbers or DOIs necessary to access your data. If you wish to make changes to your Data Availability statement, please describe these changes in your cover letter and we will update your Data Availability statement to reflect the information you provide

The literature search strategy is in Supplementary Information S1_File. The supporting information includes three additional files, which are submitted along with the manuscript. The work does not include any primary data.

**Reviewer #1:**

The aim of this study is to synthesise the literature associated with fatigue interventions to investigate the outcomes that may be applicable to ‘Long Covid’. The article reports an innovative and interesting work.

Thank you for these comments we appreciate your consideration of our work.

In the introduction I suggest to add reference to the extent of the long covid syndrome, the correct definition (with further subdivisions depending on whether the symptoms develop before or after four weeks, according to NICE guidelines) and hints of the physiopathology justifying the different treatments.

The references 17-21 include the current literature associated with Long Covid syndrome especially in ref 20, the specific range of international thinking associated with the treatment choices. We think the addition of the NICE guideline is out of place given the focus on fatigue management.

The aims of the study are well explained. The structure of the study is well organized. The materials and methods sections are discussed in details. I suggest to add in the text the initials of the author who screened the articles.

We have amended this according to your suggestion.

Statistical analysis is well conducted and appropriate.

Thank you

The results are clearly explained in the text and there are not repetitions between the text and the table. However, I recommend making the table clearer and faster to compare. At the end of each table I would insert the abbreviations used.

We have amended this according to your suggestion.

In addition, the methods explain that the selected studies were related to post-viral fatigue, in the tables, however, studies related to fibromyalgia symptoms are shown. Please clarify this important concern.

We have taken Fibromyalgia to be a post-viral syndrome see [www.healthguideinfo.com/fibromyalgia/p113865/](http://www.healthguideinfo.com/fibromyalgia/p113865/) that highlights the Epstein-Barr virus that causes a range of immunodeficiency. The identification of fibromyalgia is widely accepted to our knowledge and understanding.

In the discussion section I suggest to add reference to Maccarone MC, Magro G, Tognolo L, Masiero S. Post COVID-19 persistent fatigue: a proposal for rehabilitative interventions in the spa setting [published online ahead of print, 2021 Jun 4]. Int J Biometeorol. 2021;1-3. doi:10.1007/s00484-021-02158-1.

This reference provides a compelling and interesting treatment option for post viral fatigue but does not appear to be a good fit in the conclusion, but we will be citing in another article related to treatment modalities in sport and leisure facilities.

The work weakness has already been identified by the authors in the limitations section. In addition, making an inclusive analysis of methods of medicine recognized by the scientific community, alternative medicine methods, psychological and mind-body interventions can be a limitation because it risks confusing the reader or suggesting solutions not considered to be used at first. It would be appropriate to discuss this point further to avoid confusion.

Thanks, we have added one sentence to reflect this concern in the analysis but we see this as a strength of the paper P26

**Reviewer #2:**

Dear Authors, it has been a pleasure to review your article and the topic is of great importance in the field of rehabilitation medicine. However, in my opinion, there are some issues that should be addressed.

Thanks for your review, we have made changes as follows

1. I think, that in the aim of the study should be used “systematically” instead of “systemic”

Thank you, this is corrected on page 5.

2. Under Eligibility criteria it is said that “mixed interventions and studies involving a pharmacological component were included only when fatigue outcome measurement data could be extracted. It is not clear whether pharmacological interventions, if they were not mixed with other methods, were included or not. Were studies reporting other interventions, but not reporting fatigue outcome measurement were included? This is confusing. Could you please clarify?

We have added a statement to clarify on page 5 to say that pharma studies were excluded.

3. Could you, please, clarify the 10% of articles that was screened by the second reviewer? The reasoning behind this approach is not obvious in the text.

We have added the following 10% of the papers screened by a second review author to check for conformity of selection and to quality assess the level of agreement between reviewers

4. Results of the quality appraisal of included studies could be reported in Table 1.

We have now included MMAT quality appraisal scores in Table 1 and have also provided comments about the preliminary use of the MMAT on page 7.

5. Could you more precisely describe the expert panel and their role in the study? How many were they? Which professions? Why they can be called experts? How their input affected decisions and results?

A short additional paragraph has been added page 8.

6. Since the effect sizes has been calculated based on the outcome measures used in the included studies, it would be important to include these measures in the descriptions of studies (Table 1 or Figure2).

Effect size values have been added to Figure 2.

7. Since the information overlaps between Table 2 and Figure 2, consider omitting Table 2 from the article.

Baseline severity and effect size values have been removed from Table 3 (which has also been correctly relabelled as table 2).

8. Although, discussion includes several interesting and important aspects of fatigue treatment, it lacks the summary and interpretation of the direct results of the study.

The summary is in the results section and an additional sentence is in the discussion

9. Conclusions should contain only information that directly answers to research question(s) included in the aim of the study and are based on the results of this study. Please, revise conclusions.

Conclusion is revised in line with your comments.

**Reviewer #3:**

The authors performed a study addressing “A mixed-methods systematic review of post-viral fatigue interventions: are there lessons for long Covid?”. There is one point that needs to be corrected.

I think the Conclusion is too long. Can you make it concise? It is unclear what you want to show in this research paper.

This has been revised in line with point 9 of Reviewer 2.

________________________________________
